# Long-term survival and differentiation of retinal neurons derived from human embryonic stem cell lines in un-immunosuppressed mouse retina

**Published:** 2012-04-12

**Authors:** Dustin Hambright, Kye-Yoon Park, Matthew Brooks, Ron McKay, Anand Swaroop, Igor O. Nasonkin

**Affiliations:** 1 Neurobiology-Neurodegeneration & Repair Laboratory (N-NRL), National Eye Institute, National Institutes of Health, Bethesda, MD; 2 Laboratory of Molecular Biology and NIH Stem Cell Unit, National Institute of Neurological Disorders and Stroke, National Institutes of Health, Bethesda, MD

## Abstract

**Purpose:**

To examine the potential of NIH-maintained human embryonic stem cell (hESC) lines TE03 and UC06 to differentiate into retinal progenitor cells (hESC-RPCs) using the noggin/Dkk-1/IGF-1/FGF9 protocol. An additional goal is to examine the in vivo dynamics of maturation and retinal integration of subretinal and epiretinal (vitreous space) hESC-RPC grafts without immunosuppression.

**Methods:**

hESCs were neuralized in vitro with noggin for 2 weeks and expanded to derive neuroepithelial cells (hESC-neural precursors, NPs). Wnt (*Integration 1* and *wingless*) blocking morphogens Dickkopf-1 (Dkk-1) and Insulin-like growth factor 1 (IGF-1) were used to direct NPs to a rostral neural fate, and fibroblast growth factor 9 (FGF9)/fibroblast growth factor-basic (bFGF) were added to bias the differentiation of developing anterior neuroectoderm cells to neural retina (NR) rather than retinal pigment epithelium (RPE). Cells were dissociated and grafted into the subretinal and epiretinal space of young adult (4–6-week-old) mice (C57BL/6J x129/Sv mixed background). Remaining cells were replated for (i) immunocytochemical analysis and (ii) used for quantitative reverse transcription polymerase chain reaction (qRT–PCR) analysis. Mice were sacrificed 3 weeks or 3 months after grafting, and the grafts were examined by histology and immunohistochemistry for survival of hESC-RPCs, presence of mature neuronal and retinal markers, and the dynamics of in vivo maturation and integration into the host retina.

**Results:**

At the time of grafting, hESC-RPCs exhibited immature neural/neuronal immunophenotypes represented by nestin and neuronal class III β-tubulin, with about half of the cells positive for cell proliferation marker Kiel University -raised antibody number 67 (Ki67), and no recoverin-positive (recoverin [+]) cells. The grafted cells expressed eye field markers paired box 6 (*PAX6*), retina and anterior neural fold homeobox (*RAX*), sine oculis homeobox homolog 6 (*SIX6*), LIM homeobox 2 (*LHX2*), early NR markers (Ceh-10 homeodomain containing homolog [*CHX10*], achaete-scute complex homolog 1 [*MASH1*], mouse atonal homolog 5 [*MATH5*], neurogenic differentiation 1 [*NEUROD1*]), and some retinal cell fate markers (brain-specific homeobox/POU domain transcription factor 3B [*BRN3B*], prospero homeobox 1 [*PROX1*], and recoverin). The cells in the subretinal grafts matured to predominantly recoverin [+] phenotype by 3 months and survived in a xenogenic environment without immunosuppression as long as the blood–retinal barrier was not breached by the transplantation procedure. The epiretinal grafts survived but did not express markers of mature retinal cells. Retinal integration into the retinal ganglion cell (RGC) layer and the inner nuclear layer (INL) was efficient from the epiretinal but not subretinal grafts. The subretinal grafts showed limited ability to structurally integrate into the host retina and only in cases when NR was damaged during grafting. Only limited synaptogenesis and no tumorigenicity was observed in grafts.

**Conclusions:**

Our studies show that (i) immunosuppression is not mandatory to xenogenic graft survival in the retina, (ii) the subretinal but not the epiretinal niche can promote maturation of hESC-RPCs to photoreceptors, and (iii) the hESC-RPCs from epiretinal but not subretinal grafts can efficiently integrate into the RGC layer and INL. The latter could be of value for long-lasting neuroprotection of retina in some degenerative conditions and glaucoma. Overall, our results provide new insights into the technical aspects associated with cell-based therapy in the retina.

## Introduction

Photoreceptor death in retinal and macular degenerative diseases is a leading cause of inherited vision loss in developed countries. Novel therapeutic strategies have recently emerged, from mechanical to cell based, to repair neural circuits affected by photoreceptor (PR) cell loss [1]. Trophic factor delivery to extend the life of dying PRs has been pursued in experimental animals [2-5] and in some instances in the clinic [6]. Gene therapy approaches have been applied successfully in one type of Leber congenital amaurosis and remain viable when etiology of disease is understood and the size of a gene is not prohibitive for packaging capacity of the viral vector. Retinal implants [7,8] utilize a high-tech mechanical device placed on the retina to capture photons and transmit the electric signals to ganglion cells. Such a device is designed to replace lost PRs and has been used in the clinic with promising outcomes [9,10]. The concept of transplanting an immature retinal sheet into the subretinal space goes back to 1946 [11]. While seemingly unattainable, the approach has shown some promising outcomes [12], with the ability of such grafts to survive long-term, preserve layers, establish synaptic connectivity in the host retina, and evoke activity in the visual cortex [13,14].

Transplantation of human embryonic stem cell (hESC)- or induced pluripotent stem cell (iPS)-derived retinal progenitors or retinal neurons is a relatively recent direction for retinal therapies [15]. hESCs or iPSs can be directed to retinal fate with variable efficiency [16-21]. Compared to the retinal sheet transplantation strategy where the donor retina and its preservation constitute major limitations, the stem cell approach is based on using hESCs or iPSs, which provide an unlimited source of cells. Furthermore, there is optimism stemming from research on mouse ESCs that such human protocols might be improved by engineering the development of the whole retina in a dish [22].

The transplantation of retinal cells into mammalian retina produces variable outcomes and success. A greater understanding of biology and improvements in methodology are required before such protocols may be introduced into the clinic [19,23-27]. Some of the key obstacles in transplantation studies include immunological compatibility of graft and host [27,28], the outer limiting membrane (OLM) being a barrier for retinal integration [25,26,29], and glial cells/glial scar at transplantation site preventing efficient integration [19,26,30,31]. The retinal stem cell-based approach requires that transplanted cells migrate into the retinal layer(s) affected by genetic lesion, undergo terminal maturation, acquire the appropriate cell fate, and establish needed synaptic interactions. This is different from other systems, such as transplantation of insulin-producing β-cells [32], skin cells [33], or blood cells [34], which require the newly grafted cells to primarily acquire the proper postmitotic cell fate. Although biology of specific synaptic connectivity during retinal development is still poorly understood, promising reports indicate the feasibility of this direction, and such an approach, still largely heuristic in nature, may at least partially alleviate blindness in experimental animals with PR degeneration [29,35-37]. The progress in cell-sorting techniques [38,39] based on cell-surface antigens rather than fluorescent markers gives further hope of generating a defined cell population for cell replacement.

Transplantation is traditionally accompanied by immunosuppression, which has detrimental side effects, such as tumorigenesis [40,41], and may contribute to regenerative processes in the central nervous system (CNS) [42,43], thus masking the therapeutic effect exerted by neural graft. The retina and brain are reported to be partially immunoprivileged sites [31,44,45]. However, survival of neural grafts in subretinal space with and without immunosuppression still varies [5,27,46-50], requiring a more systematic examination. Interestingly, the transplanted neural progenitors themselves may exert an immunomodulatory effect on the host CNS [51]. Allogeneic mouse retinal grafts can also undergo apoptosis in degenerating mouse retina [24].

This study was initiated to examine the survival and integration of hESC-derived neural progenitors that were transplanted into normal adult mouse retina with no immune suppression. We show that xenogenic human grafts comprising of postmitotic hESC-RPCs carrying PR markers can survive in adult mammalian retina for up to 12 weeks with no signs of deterioration. We also report that hESC-RPCs can integrate from the epiretinal grafts into host’s RGC layer and inner nuclear layer (INL) but not PR layer. The cells from the subretinal grafts, however, show limited integration into the PR layer and only when retina was damaged during transplantation. We also noted the instructing role of the subretinal but not epiretinal niche in promoting further maturation of grafted cells to PRs. Taken together, our data may help in refining protocols of hESC-derived retinal cell transplantation.

## Methods

### Animals

Young adult C57BL/6Jx129/SvJ wild-type mice (4–6-weeks old; the Jackson Laboratory, Bar Harbor, Maine) were used for transplantation experiments. All animals were housed and treated in accordance with the ARVO Statement for the Use of Animals in Ophthalmic and Vision Research and in accordance with the standards issued by the NIH animal facilities, NIH approval #ASP 08–610.

### Human embryonic stem cells

hESCs were maintained and supplied by the NIH Stem Cell Unit. TE03 (Technion-Israel Institute of Technology, Haifa, Israel) and UC06 (University of California San Francisco, San Francisco, CA) were separately differentiated and used for grafting.

### Cell culturing and differentiation factors

Neurobasal medium with B27/N2 supplementation (all from Invitrogen, Grand Island, NY) and plastic Petri dishes (Corning Inc., Corning, NY) covered with 0.1% gelatin (Sigma-Aldrich, St. Louis, MO) were used for cell culture during differentiation. Noggin, Dkk-1, IGF-1, FGF9 were from R&D Systems, Minneapolis, MN, and bFGF was from Sigma-Aldrich, St. Louis, MO.

### Cryosectioning and slides

The Microm HM550 cryostat (Thermo Scientific, Rockville, MD) was used to produce 16-μm serial sections of mouse eyes. Microscope slides were purchased from Fisher Scientific (Pittsburg, PA). Glass coverslips were purchased from Brain Research Laboratories (Newton, MA).

### In vitro differentiation

hESC lines were grown on a mouse embryonic fibroblast feeder layer (CF1 strain, The Jackson Laboratory, Bar Harbor, Maine) according to the protocols described (NIH research). The colonies (day 4–5 after passage) were dislodged and placed on gelatin-coated Petri dishes and cultured at high density in Neurobasal medium supplemented with 1x B27 without retinoic acid, 1x N2, 1x penicillin–streptomycin antibiotic mix, L-glutamine (1%), Minimal Essential Medium nonessential amino acid solution (1%; all from Invitrogen), BSA fraction V (0.1%), β-mercaptoethanol (0.1 mM; both from Sigma-Aldrich), and recombinant noggin (100 ng/ml; R&D Systems) but without bFGF to induce neuralization [18]. At 14 days of differentiation, the cultures were supplemented with bFGF (10 ng/ml) [19], and 50% of the media was renewed every other day. At day 28, neural rosettes were excised mechanically and replated as large clusters of neuroepithelial cells (hESC-NPs) [6] on gelatin/laminin-coated plates and cultured at high density (95%–100% confluency). Wnt-blocking morphogens Dkk-1 and IGF-1 (10 ng/ml each) were applied for 1 week immediately after replating to anteriorize cells to a rostral neural fate [12]. Cells were then cultured further with the addition of FGF9 and bFGF (both at 10 ng/ml) as well as noggin until grafting to bias cells to an NR rather than an RPE cell fate [13-15]. At day 50, hESC-RPCs were dissociated with cell dissociation buffer (Invitrogen) and trypsin-like enzyme (Invitrogen) and suspended in Neurobasal medium at ~50×10^3^ cells/μl for transplantation.

### Immunocytochemical and quantitative reverse transcription coupled polymerase chain reaction analysis of cells

Immediately after transplantation, some of the remaining cells were replated to evaluate the viability and differentiated state of transplanted cells. Antibodies against human nuclei (HNu), human nestin, recoverin, Kiel University-raised antibody number 67 (Ki67), doublecortin (DCX), and neuronal class III β-tubulin (Tuj1) were used for this analysis. Immunohistochemistry was done as described previously [6]. Total RNA was prepared from (i) undifferentiated hESC cells and (ii) cells at day 50 (transplantation) using RNeasy Mini kit (Qiagen, Valencia, CA) according to the manufacturer’s protocol; briefly cells were first lysed and then homogenized, the lysates were then loaded onto the RNeasy silica Mini spin columns, and after RNA was bound to silica gel, all contaminants were washed away, and pure concentrated RNA was eluted in water. RNA was then converted to cDNA with Superscript II (Invitrogen, Carlsbad, CA), and used for quantitative reverse transcription polymerase chain reaction (qRT–PCR) analysis on an ABI 7900HT Fast Real-Time PCR System (Applied Biosystems, Foster City, CA) with SYBR Green Master Mix (Applied Biosystems). Oligonucleotide primers (Table 1) specific to (i) pluripotent hESCs (octamer-binding transcription factor 3/4 [*OCT3/4*], *NANOG*, sex-determining region Y gene-related high mobility group box 2 [*SOX2*]), (ii) markers of the anterior neuroectoderm (forkhead box protein G1 [*FOXG1*], [*SIX3*], sine oculis homeobox homolog 6 (Drosophila) [*SIX6*], [*LHX2*), (iii) markers of the eye field (*PAX6, SIX3, SIX6, RAX* [*RX*]), (iv) retinal progenitors (*CHX10, MASH1, NEUROD1*), RPE (microphthalmia-associated transcription factor, *MITF*), PRs (recoverin, cone-rod homeobox gene [*CRX*], neural retina-specific leucine zipper, [*NRL*]), RGCs (*MATH5, BRN3B*, insulin gene enhancer protein [*ISL1*]), and horizontal neurons (*PROX1*) were used for qRT–PCR analysis, which was performed in triplicate at both time points (“undifferentiated hESCs” and “day 50, grafting”). The qRT–PCR data were analyzed using the ΔΔCt method (as described in Livak KJ, Schmittgen TD. Methods 2001 paper), with geometric averaging of β-actin (*ACTB*) and glyceraldehyde-3-phosphate dehydrogenase (*GAPDH*) as the endogenous controls (outlined in Vandesompele J. et al., Genome Biol 2002). Briefly, the standard approach of DNA quantification by real-time qRT–PCR is based on plotting measured fluorescence (in our case SYBR Green I cyanine dye incorporated into DNA during PCR) against the number of PCR cycles on a logarithmic scale. During the exponential phase of qRT–PCR, when reagents are not limited, the amount of cDNA (target) is assumed to be doubling every cycle. First, ΔCt analysis is done, which takes the Ct (cycle number, or cycle threshold) value for the gene of interest, divided by Ct of a housekeeping gene in the same sample, at the point when the signal just becomes detectable above the background and the amplification is in exponential phase; the log2 difference is then generated. The more abundant the mRNA for gene X is, the quicker this point is reached, thus giving earlier Ct values and allowing to quantitatively evaluate the expression of gene X. In our case Ct values were generated automatically using SDS2.3 software (Life Technologies, Carlsbad, CA). Second, ΔΔCt analysis is done, where ΔΔCt equals ΔCt [sample] (Ct value for “day 50” sample normalized to the endogenous housekeeping gene) minus ΔCt [reference] (Ct value for “undifferentiated hESCs also normalized to the endogenous housekeeping gene). Collectively, ΔΔCt method is a normalization procedure, which allows comparison of gene expression levels in different RNA samples by taking into account the differences in quality and total amount of RNA in samples. Expression levels at day 50 (grafting) were presented as log2 values of the expression level differences compared to that found in undifferentiated hESCs. The analysis was done in technical triplicates, with one biological replicate.

**Table 1:** qRT–PCR primers.

**Primers (5′-3′)**	**Gene name**
F: AGATGCCTCACACGGAGACT	*NANOG*
R: TTTGCGACACTCTTCTCTGC	
F: TGAGTAGTCCCTTCGCAAGC	*OCT3/4*
R: GCGAGAAGGCAAAATCTGA	
F: GGGGGAATGGACCTTGTATAG	*SOX2*
R: GCAAAGCTCCTACCGTACCA	
F: CCGGAAGACAGGATACAGGT	*CHX10*
R: ACTCCGCCATGACACTGC	
F: TTTGAGTTACAACGGCACCA	*FOXG1*
R: TCTGAGTCAACACGGAGCTG	
F: CCAAGGACTTGAAGCAGCTC	*LHX2*
R: AAGAGGTTGCGCCTGAACT	
F: CAGGTGCCGATGGAAGTC	*MITF (RPE‐SPECIFIC ISOFORM)*
R: GCTAAAGTGGTAGAAAGGTACTGCTT	
F: TCACCATGGCAAATAACCTG	*PAX6*
R: CAGCATGCAGGAGTATGAGG	
F: TTCGAGAAGTCCCACTACCC	*RAX*
R: ACTTAGCCCGTCGGTTCTG	
F: CTCCTCCCCCACTCCTTC	*SIX3*
R: GGGTATCCTGATTTCGGTTTG	
F: GGACACTGCAAGCCCAGTAT	*SIX6*
R: ATGATTCGCGCCCTTTCT	
F: CGACTTCACCAACTGGTTCTG	*MASH1*
R: ATGCAGGTTGTGCGATCA	
F: CAGACCTATGGACGCAATCA	*MATH5*
R: CAACCCATTCACAAGATCCA	
F: CCCTTTGAACCCCACCTC	*BRN3B*
R: CTTCCTGCAAACAGCCATCT	
F: CGAGTTGGTACACACCGTCA	*CRX*
R: TCTCTTCACATCTCGCCTTTC	
F: AAGGACAAGAAGCGAAGCAT	*ISL1*
R: TTCCTGTCATCCCCTGGATA	
F: CTGCTCAGGACCTACTAACAACAA	*NEUROD1*
R: GTCCAGCTTGGAGGACCTT	
F: TCCTCTCGGCCATTTCTG	*NRL*
R: CTCAAACTTCATCAAGTCAAAGTCA	
F: AAATATCACCTTATTCGGGAAGTG	*PROX1*
R: TTTTCAAGTGATTGGGTGACA	
F: TAACGGGACCATCAGCAAG	*RCVRN*
R: CCTCGGGAGTGATCATTTTG	
F: GCAACTACGTGGGCGACT	*TUBB3*
R: CGAGGCACGTACTTGTGAGA	

### Subretinal transplantation

Transplantation equipment included a nano-injector (World Precision Instruments, Sarasota, FL), pulled glass micropipettes (Drummond Scientific Company, Broomall, PA), 29G insulin syringe (Becton Dickinson & CO, Franklin Lakes, NJ), and a dissection microscope (SZ61; Olympus, Center Valley, PA). General anesthesia was used during transplantation and included a mixture of ketamine (87 mg/kg) and xylazine (10 mg/kg) administered intraperitoneally at 0.1 ml/g bodyweight. Once properly sedated, the animals were placed under the dissection microscope; the eyes were covered with a thin film of mineral oil to prevent drying, and a small incision was made in the cornea using a sharp insulin syringe. Using the nano-injector, the blunt-ended tip of a micropipette filled with cells was guided through the incision in the cornea and advanced trans-retinally until it met resistance due to rigid choroid/scleral tissue. The needle was then slightly withdrawn, and as it was very slowly being pulled out to create space for grafted cells, hESC-RPC suspension (≤1.5 μl, total of about 50,000 cells) was slowly deposited into the subretinal space. This transplantation methodology, due to a mouse NR being so thin, inevitably left about 20% of grafted cells epiretinally, adjacent to the RGC layer. Both eyes were injected for each animal. For the TE03 hESC line, 26 subretinal grafts (13 animals) were generated; six animals (12 eyes) were analyzed at 3 weeks and seven animals (14 eyes) were analyzed at 3 months after grafting. For the UC06 hESC line, 14 subretinal grafts (seven animals) were generated; three animals (six eyes) were analyzed at 3 weeks and four animals (eight eyes) were analyzed at 3 months after grafting.

### Enucleation, fixation, and embedding of eyes for sectioning

Enucleation of the eye was done with a fine-point microdissection forceps and fine-point microdissection scissors (Electron Microscopy Sciences, Ft. Washington, PA). Eyes were fixed in paraformaldehyde (4%, Sigma-Aldrich) for 5 min, rinsed with 1x PBS (KD Medical, Columbia, MD; NaCl 90 g/l; Na_2_HPO_4_, anhydrous, 7.10 g/l; KH_2_PO_4_, 2.3g/l; UltraPure Water), cryoprotected in 20% and then 30% sucrose, and then snap frozen in OCT embedding material (Tissue-Tek, Torrance, CA) and serially sectioned at 10 μm.

### Histological staining

For cresyl violet (CV) staining, serial sections were sequentially washed with PBS and deionized water, stained with CV for about 1 min, dehydrated with increasing concentrations of ethanol, mounted with DPX solution (Sigma-Aldrich), and examined with a light microscope for the presence of hESC-RPC grafts.

### Immunohistochemistry

Immunohistochemistry (IHC) staining was performed using primary antibodies (Table 2) for human nuclei (HNu), human nestin, DCX, Tuj1, recoverin, rhodopsin, glial fibrillary acidic protein (GFAP), human synaptophysin, Ki67, and ionized calcium-binding adaptor molecule-1 (Iba-I).

**Table 2:** Primary antibodies.

**Target phenotypes**	**Target proteins/ Epitopes**	**Host**	**Dilution**	**Vendor**
**Primary antibodies**				
Neural stem cell and/or precursor	Nestin (human, not rat/mouse-specific)	Mouse	1:400	Millipore
Neuronal precursor	Doublecortin (DCX)	Guinea pig	1:3,000	Millipore
Neuron - early	Type III b-Tubulin epitope J1 (Tuj1)	Rabbit	1:1,000	Covance
Neuronal precursor	Doublecortin-like kinase (DCAMKL1)	Rabbit	1:50	Dr. Walsh, Harvard
Mitotic marker	Ki67 antigen (NCL-Ki67p), Kiel University -raised antibody # 67	Rabbit	1:1,000	Novocastra Labs
Human Nuclei -all	Human nuclear protein epitope (HNu)	Mouse	1:1,200	Millipore
Neuron -human	Synaptophysin, human, not rat/ mouse –specific(Syn)	Mouse	1:800	Millipore
Muller glia -activated	GFAP	Rabbit	1:700	DAKO
Photoreceptor	Recoverin (Rec)	Rabbit	1:1,000	Millipore
Rod photoreceptor	Rhodopsin, Rho4D2	Mouse	1:100	Dr.Molday (UBC)
Microglia	Ionized calcium binding adaptor molecule-1 (Iba-1)	Rabbit	1:200	Waco Chemicals USA
**Secondary antibodies**				
Alexa Flour 488	Goat anti-Rabbit	Goat	1:400	Invitrogen
Alexa Flour 594	Goat anti-Mouse	Rabbit	1:400	Invitrogen

**Figure 1:**
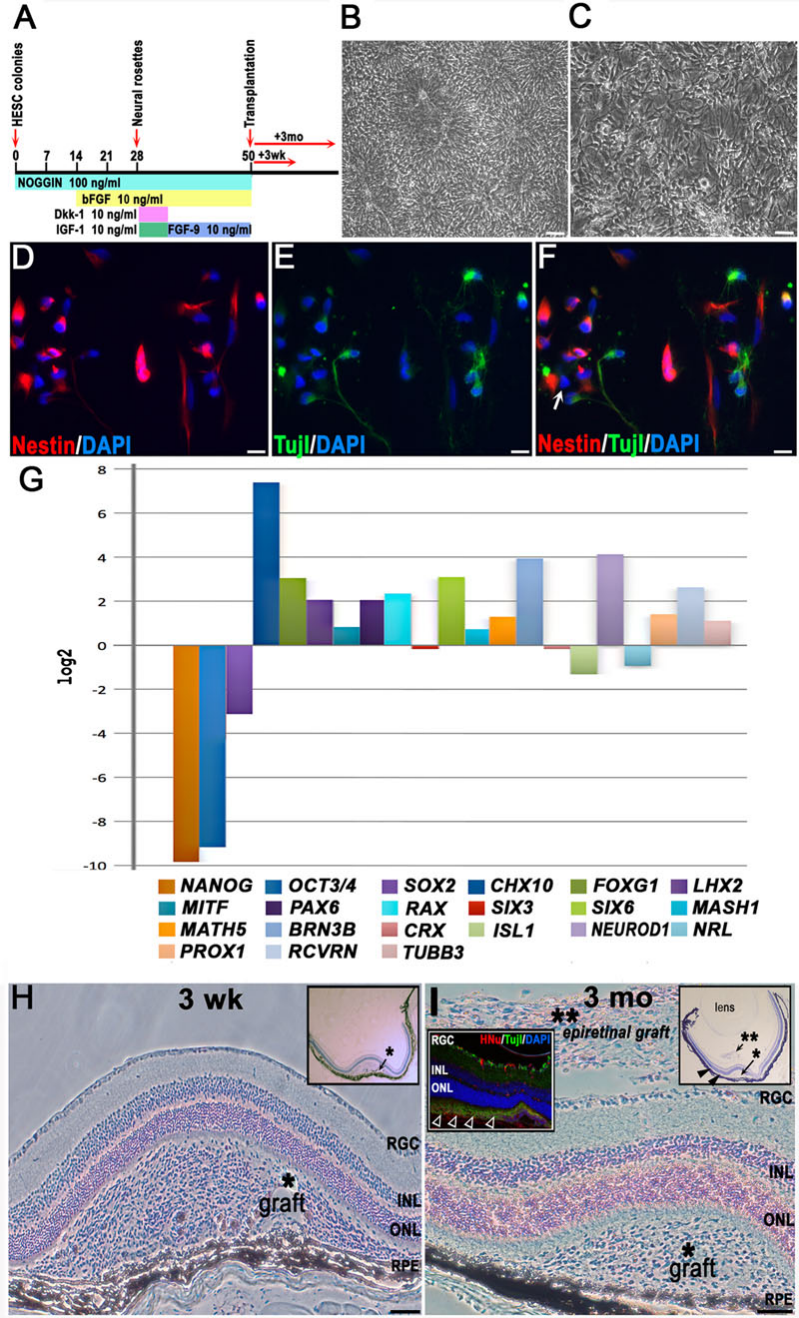
Human embryonic stem cell retinal differentiation diagram, characterization of cells for ocular grafting, and graft identification by histological analysis. **A**: For differentiation, human embryonic stem cells (hESCs) were neuralized by withdrawal of basic FGF (bFGF) and providing noggin morphogen at 100 ng/ml in adherent hESC cultures. Noggin was kept in adherent rosette cultures throughout the differentiation procedure. bFGF was applied at day 14 and was kept thereon until grafting. At day 28, rosettes were excised mechanically from the cultures and replated to start a passage-1 culture of pure neural rosettes; Wnt-inhibiting morphogens Dkk-1 and IGF-1 (10 ng/ml each) were added and were kept for 1 week to direct the early neuroectoderm toward early retinal fate. From day 35 to day 50 (grafting), the late neural rosettes were kept in dense cultures in Neurobasal medium supplemented with 1x B27/N2 and bFGF+FGF9 (10 ng/ml each) to bias early retinal cells toward a neural retina rather than an RPE cell fate. **B**: These are neuralized hESCs (neural rosettes), shown at day 28 after initiation of neural differentiation protocol (the scale bar represents 50 μm). **C**: These are early neurons differentiating from late neural rosettes harvested for cell transplantation on day 50 and replated at high density (the scale bar represents 50 μm). **D**-**F**: These are cells replated immediately after transplantation at low density and analyzed the next day. The cells display neural (nestin) or early neuronal (Tuj1) immunophenotypes with only rare cells (less than 1%) displaying nestin [-] Tuj1 [-] immunophenotype (shown with a white arrow in [**F**]), (the scale bar represents 10 μm). **G**: This is quantitative RT–PCR analysis of hESC-derived retinal progenitors prepared for transplantation at day 50 of retinal differentiation protocol; *β-ACTIN* and glyceraldehyde-3-phosphate dehydrogenase (*GAPDH*) served as the housekeeping genes for normalization. The analysis was done in technical triplicates, with one biologic replicate, therefore no error bars are shown. Expression level values for all genes at day 50 (grafting) are presented as the binary logarithm (log_2_) values (fold change) using comparative ΔΔCt method. In this plot “0” (i.e., log_2_ [1]) represents no change in gene expression at day 50 (compared to that at day 0, in undifferentiated hESCs). Markers of pluripotency, *NANOG, OCT3/4*, and *SOX2*, are downregulated in human embryonic stem cell-derived retinal progenitor cells (hESC-RPCs) at day 50, while a forebrain progenitor marker *FOXG1*, several eye field markers (*RX* (*RAX*), *PAX6, LHX2, SIX6*), early neural retinal progenitor markers (*CHX10, MASH1, MATH5, NEUROD1*), photoreceptor marker recoverin (*RCVRN*), retinal ganglion cell (RGC) marker (*BRN3B*), and horizontal marker (*PROX1*) show upregulation. The retinal pigment epithelium-specific isoform of *MITF* shows only a slight upregulation, indicating that the cells were induced toward neural retina rather than an RPE fate. **H**: Characteristic large subretinal graft found at 3 weeks following cell transplantation, cresyl violet (CV) staining. Major retinal cell layers and RPE are indicated. The asterisk shows the likely needle track from injection, which has several separated RPE cells embedded into the graft cell mass but overall caused little damage to the RPE. The inset shows an overview of a mouse eye carrying such a graft (the scale bar represents 100 μm). **I**: Typical large subretinal graft surviving for 3 months after transplantation, CV staining. Cells left on top of the RGC layer during needle withdrawal formed epiretinal grafts (**), which were found frequently in sections, and showed no tumors but persisted in a less differentiated state. The right inset shows the overview of a mouse eye section carrying such a graft. The closed arrowheads show a graft spreading within the subretinal space. The inset on the left is a fluorescent image showing the spreading of grafted HNu [+] Tuj1 [+] hESC-RPCs in the subretinal space (open arrowheads) at 3 months (the scale bar represents 100 μm).

Eye sections demonstrating the presence of grafted cells by CV (Figure 1H,I) were sequentially incubated with 0.1% Triton X-100/PBS (PBS-T) at room temperature for 30 min, followed by 1 h incubation in blocking solution (5% pre-immune serum and 0.1% PBS-T) at room temperature, and then incubated with primary antibodies diluted in blocking solution at 4 °C overnight. HNu antibody was used to identify grafted human cells [18,21]. Ki67 antibody was used to examine the mitotic activity of cells [22]. Human nestin antibody was used to identify multipotential human neural precursor cells [23]. Tuj1 antibody was used to identify neuronal cells. DCX antibody was used as a marker for neuroblasts and young neurons [24]. Recoverin and rhodopsin antibodies were used as PR markers [25]. Human synaptophysin antibody was used to identify the presynaptic part of human boutons established by maturing hESC-RPCs [6]. GFAP antibody was used as a marker for activated Müller glia cells [26]. Iba-1 was used to identify microglia cells [27]. DAPI staining was used to identify nuclei of any cell type. Following overnight incubation with primary antibodies, sections were washed three times with 0.1% PBS-T and then incubated with the corresponding secondary antibodies (Alexa Fluor 594 goat antimouse, Alexa Fluor 488 goat antirabbit) at room temperature for 45 min. The slides were washed three times with 0.1% PBS-T solution, incubated with 4', 6-diamidino-2-phenylindole (DAPI) solution (1 μg/ml) for 10 min, and then washed again with 0.1% PBS-T solution. For negative controls, slides were treated similarly except that primary antibodies were omitted. The specimens were mounted with ProLong Gold Antifade medium (Invitrogen) and examined using an Olympus (Center Valley, PA) epifluorescent microscope IX51 with a Spot (Sterling Heights, MI) CCD Camera RT3 and Leica (Buffalo Grove, IL) SP2 confocal microscope. For high-resolution confocal microscopy, *z*-series of images (with a *z*-step of 0.2 μm, 15–20 optical sections) were collected using a 63x1.32 numerical aperture oil immersion objective (Leica SP2). Consecutive optical planes (*z* series) of selected fields were analyzed to evaluate distribution and co-localization of fluorescent signals, with subsequent virtual resectioning at the *x* and *y* axes.

### Statistical analysis

Data on human RPC grafts at 3 weeks and 3 months were obtained from serial sections and evaluated by the StatView program (Abacus Corporation, Baltimore, MD). The difference in Tuj1 and recoverin expression between TE03 and UC06 grafts was minimal at 3 weeks and 3 months. Thus, results were grouped for two hESC lines for each time point and plotted as a mean of the percentages of HNu – positive ([HNu [+]) human cells carrying Tuj1 or recoverin in grafts, with corresponding standard error of the mean (SEM). Comparison of the statistical significance between expression of Tuj1 and recoverin in the subretinal space versus the epiretinal (vitreous) space was calculated with an unpaired Student *t* test (with p<0.05 considered statistically significant) after converting the percentage values to arc sin values [52].

## Results

### Differentiation of human embryonic stem cells to retinal cells

We used noggin in the absence of bFGF mitogen for 2 weeks to neuralize hESCs, as described [53] (Figure 1A). The detailed protocol is outlined in Methods, in vitro differentiation section of this paper. At day 28, the plates with differentiating hESC colonies were 95% confluent, and about one-third of each plate area consisted of neural rosettes (Figure 1B). These rosettes were isolated mechanically as described [53] (briefly, excised with a fine fire-polished and sealed pulled glass pipette), replated on gelatin/laminin-coated plates, and induced to a rostral neural tube cell fate by Wnt blocking morphogen Dkk-1 and IGF-1 for 1 week. Following retinal induction, the cells (hESC-RPCs) were cultured with FGF9 and bFGF until transplantation. Immediately after transplantation, the remaining cells were replated and evaluated by immunocytochemistry (ICC) the following day (Figure 1F).

We noted an efficient neuralization of hESCs by day 28 and downregulation of pluripotency markers and upregulation of neural and retinal markers by IHC and/or qRT–PCR by day 50 of our differentiation protocol (Figure 1A). About half (52.7%) of the cells were mitotically active as judged by Ki67 positivity (not shown). The majority (64.2%) were human nestin - positive (human nestin [+]; Figure 1D,F), and 39.8% were Tuj1 [+] (Figure 1E,F; averaged for both hESC lines). No recoverin [+] or rhodopsin [+] cells were detected at this stage by ICC. Less than 1% of cells were human nestin [-] and Tuj1 [-]. Pluripotent hESCs immediately before the differentiation protocol and hESC-RPCs at day 50 (grafting) were used for total RNA preparation and qRT–PCR. Pluripotency markers *NANOG*, *OCT3/4*, and *SOX2* were downregulated at day 50, while the eye field and NR progenitor markers, such as *RX, SIX6, PAX6, CHX10, NEUROD1*, early PR marker recoverin, and pan-RGC marker *BRN3B*, showed substantial upregulation. The RPE-specific isoform of MITF showed only a slight upregulation (Figure 1G).

### Survival and morphology of subretinal grafts

**Figure 2:**
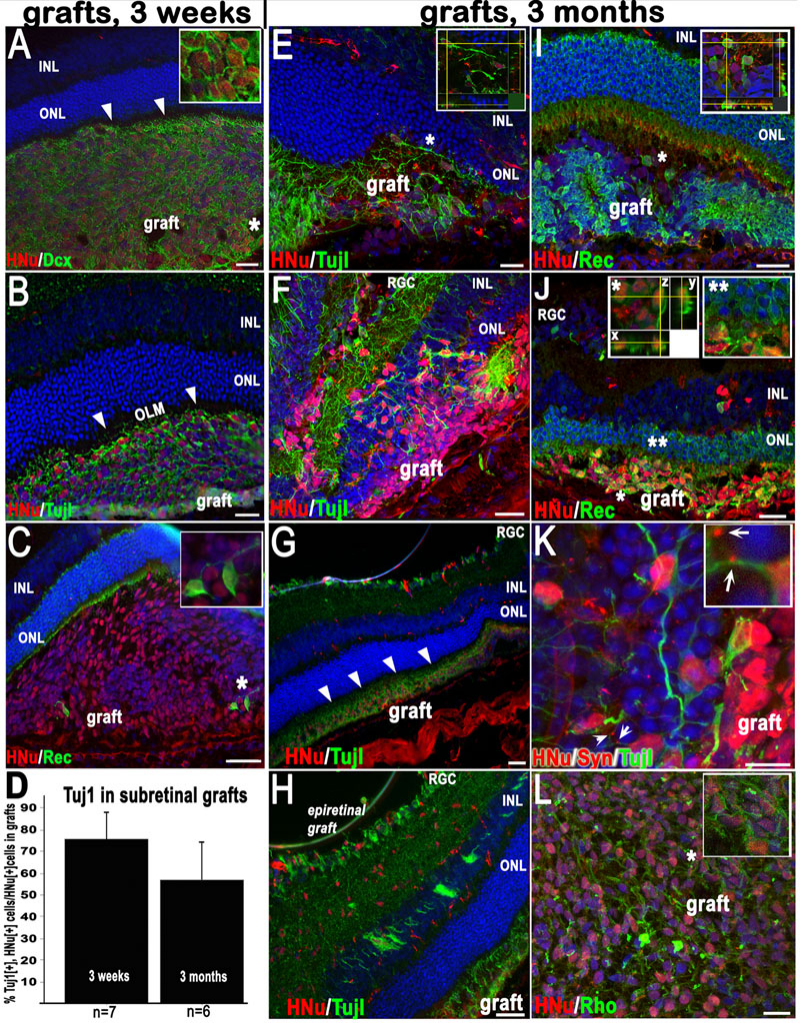
Limited retinal integration but substantial maturation of human embryonic stem cell-derived retinal progenitor cells in subretinal grafts. Immunostaining for **A**; doublecortin (DCX) and human nuclei (HNu), **B**; neuronal class III β-tubulin (Tuj1 epitope) and HNu, and **C**; Photoreceptor (PR) marker recoverin and HNu in subretinal grafts. The asterisks indicate the areas shown in insets. Note the border (indicated with closed white arrowheads) demarcating the outer nuclear layer (ONL) and the grafts in **A** and **B** showing the location of the outer limiting membrane (OLM). Subretinal grafts consisted of mostly early neuronal cells (~76%), as shown in **D**. **D** represents grouped data for both human embryonic stem cell (hESC) lines. Error bars indicate the standard error of the mean (SEM), (the means did not differ significantly between the two hESC lines by an unpaired Student’s *t*-test). The Tuj1 marker is still heavily present in subretinal grafts at 3 months (**E**-**H**); however, the cells fail to migrate into the host’s ONL and remain in the subretinal space (**E**, **H**) unless the neural retina (NR) is damaged by injection (**F**). As in **A** and **B**, the host’s OLM in the 3-month grafts presents a barrier for subretinally grafted cells to migrate into the host’s ONL (**G**, shown with closed white arrowheads), although many grafts spread over a large subretinal area (also in **G**). **H**: Cells from the epiretinal grafts integrate into the host’s retinal ganglion cell (RGC) layer, inner plexiform layer, and inner nuclear layer (INL). **I**-**L**: Mature immunophenotypes, such as recoverin (**I**, **J**), synaptophysin (**K**), and rhodopsin (**L**), are observed in these grafts. In **I** and **J**, subretinal grafts predominantly display recoverin-positive (an early photoreceptor) immunophenotype (shown in *z*-sections, see insets in **I** and **J**). The asterisks point to areas within the main images shown in these insets. In **I** and **J** we compare the integration of early hESC-derived photoreceptors into the host ONL in intact, nondamaged retinas (**I**) versus that, where retinas were damaged by the injection needle (**J**). Compare **I**, no integration of HNu [+] cells in the host’s ONL can be seen, versus **J**, where Rec (recoverin) [+], HNu [+] PRs are found embedded into the host’s ONL (indicated with two asterisks). Also see the respective inset (** in **J**). In **K** note sparse human synaptic boutons (shown with white arrows and enlarged in inset) stained with human-specific synaptic marker synaptophysin (Syn) found both on graft- (shown in inset) and host-specific neurons. The asterisks point to areas within the main images shown in the insets. The main images in the panel are confocal images. The insets show confocal *z*-stack analysis of selected areas done with virtual resectioning along the *x* and *y* planes. Scale bars: 20 μm (**A**, **B**, **E**, **F**, **I**, **J**, **L**); 50 μm (**C**, **G**, **H**); 10 μm (**K**).

**Figure 3:**
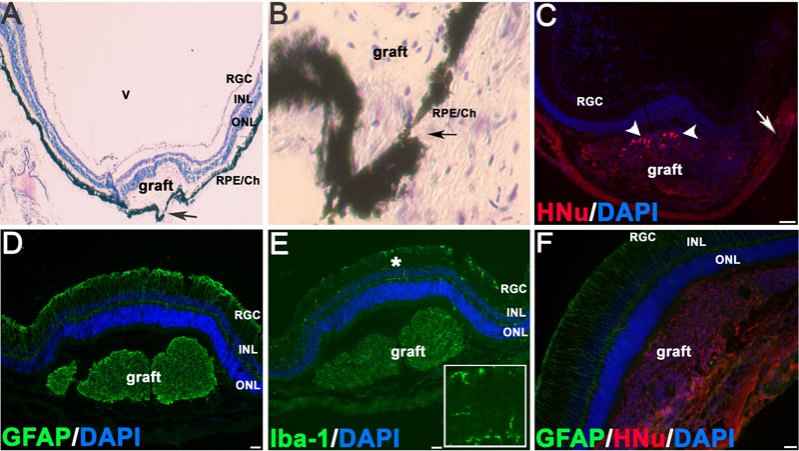
The example of grafts, which did not survive due to damage done to retinal pigment epithelium/choroid layers. **A**: This is a three-week-old subretinal graft with a typical neural retinal bulge but no human nuclei –positive (HNu [+]) cells. “V” is the vitreous space. The arrow points to the damage done to retinal pigment epithelium/choroid tissue (RPE/Ch), which likely causes the rupture of the blood-retinal barrier and exposes the xenogenic graft to the host’s immune system. **B** is the enlarged area shown in **A**, where RPE/choroid tissue is damaged (arrow). **C**: This is a three-week-old human embryonic stem cell-derived retinal progenitor cell graft with only few surviving HNu [+] cells left (white arrowheads); the RPE/choroid tissue is also damaged (white arrow). Panels **D**-**E** show the immunohistochemistry data done on retinal sections carrying the graft displayed in panel **A**. In panel **D** we demonstrate the accumulation of glial fibrillary acidic protein (GFAP), an early indicator of retinal distress, in the host retina. In panel **E** we show that microglia/macrophage marker ionized calcium binding adaptor molecule 1 (Iba-1) (known to be upregulated during the activation of these immune cells) is heavily present inside the graft and in the host retina around the grafted area. The asterisk shows the area in the main image, enlarged in the inset. The inset depicts several Iba-1 - positive cells in the host neural retina, with a typical microglial morphology. The scale bar used in panels **C**-**E** is 100 μm. **F**: This is a surviving 3-week-old subretinal graft shown for comparison; note the GFAP activation in the host retina above the graft, which did not affect the survival of such graft. The scale bar used in panel **F** is 50 μm. Abbreviations used in this legend are the following: ONL, outer nuclear layer; INL, inner nuclear layer; RGC, retinal ganglion cell (layer).

Serial CV staining of grafts at 3 weeks after transplantation showed surviving transplanted cells clustered around the transplantation site (Figure 1H). At 3 months more cells were found spreading within the subretinal space (Figure 1I, also see Figure 2G). Successful subretinal grafts (Figure 1H,I) were observed in about 25% of transplanted eyes (n=3–4 grafts/hESC line). Many surviving grafts (n=14 examined) were detected in the epiretinal area. In subretinal grafts, there was a distinct border between the graft and the outer nuclear layer (ONL) separated by the OLM (Figure 2A,B,G, arrowheads). However, in cases where the host retina had been damaged during transplantation, we observed some HNu [+] Tuj1 [+] cells, and by 3 months we also observed HNu [+] recoverin [+] neurons integrating into the ONL (Figure 2F,J). The survival of xenogenic human grafts was best when the host RPE/choroid was not damaged, as evaluated by CV staining (as in Figure 1H,I). Sections that displayed damage to the RPE/choroid (Figure 3A-C, arrows) displayed few or no surviving HNu [+] cells and strong GFAP activation [54] and microglial cell accumulation [55] in and around the grafted area.

**Figure 4:**
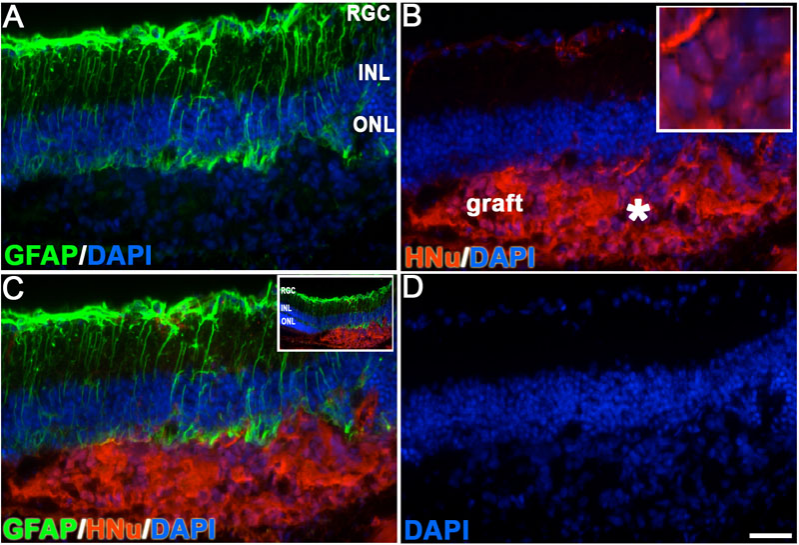
Glial fibrillary acidic protein (GFAP) activation in the host retina around the grafting site 3 weeks after transplantation. GFAP activation (**A**) was found in all examined cases where the subretinal grafts were found, regardless of whether the grafts survived or not. In the case shown, the release of human nuclei –positive (HNu [+]) immunoreactivity was found in the grafting site outside of the nuclei, indicating the initial stage of graft destruction (**B**, **C**). Inset in **C** shows a low-power image of the same graft from which the main panel was derived. Panel **D** displays the staining of nuclei of both human and mouse cells with 4', 6-diamidino-2-phenylindole (DAPI). The scale bar used in panels **A**-**D** is 50 μm. The outer nuclear layer (ONL) around the grafting site was damaged by the needle. The asterisk indicates the area shown in the inset. Abbreviations used in this legend are the following: INL, inner nuclear layer; RGC, retinal ganglion cell (layer).

**Figure 5:**
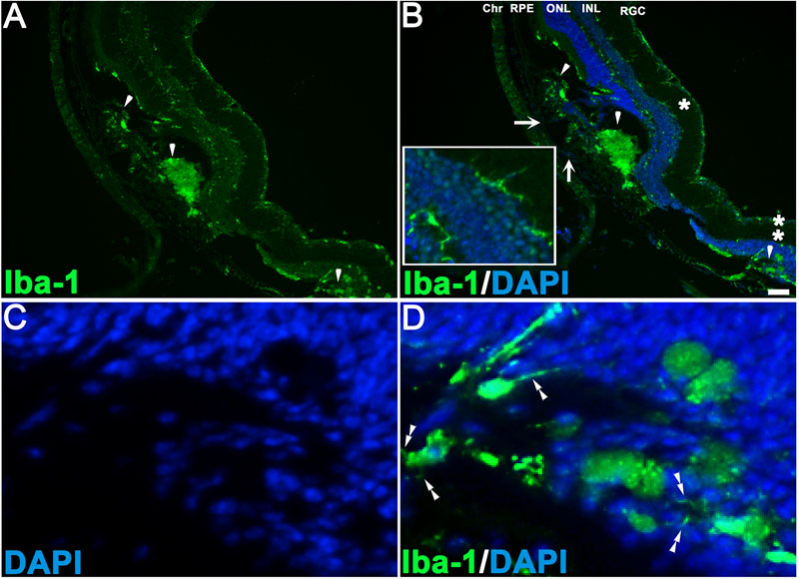
Microglia accumulation in a subretinal graft with damaged retinal pigment epithelium/choroid. This is a typical staining pattern (**A**, **B**) observed in grafts where a needle penetrated retinal pigment epithelium (RPE) and disrupted choroid (Chr) vasculature (solid white arrows in **B**), leading to the rupture of the retinal–blood barrier and exposure of xenogenic (human) graft to the host’s immune system. By 3 weeks after subretinal transplantation, there are typically no surviving human neurons in such grafts, yet some human nuclei-positive immunoreactivity occasionally may be found. Solid white arrowheads point to the accumulation of ionized calcium binding adaptor molecule 1 (Iba-1) staining where the grafted cells were placed. The area of the main image displayed in the inset in panel **B** is indicated with an asterisk (*). The inset shows several Iba-1-positive cells with a morphology typical for activated microglia. The scale bar used in panel **B** is 50 μm. Double asterisk (**) in panel **B** indicates the area, enlarged in panels **C** and **D**. This is the host photoreceptor layer with high microglial activity, where human retinal progenitors were earlier grafted but did not survive. Microglial processes are shown with double white arrowheads. The following abbreviations were used in these panels: ONL – outer nuclear layer, INL, inner nuclear layer, RGC- retinal ganglion cells, DAPI – 4', 6-diamidino-2-phenylindole.

Some grafts demonstrated slower cell degradation evident by the release of human nuclear proteins into the subretinal space, weak nuclei HNu antibody staining, and HNu [+] immunoreactivity outside the grafted cells. Such grafts also had strong activation of GFAP around, but not inside, the grafted area (Figure 4). Iba-1 immunoreactivity was prominent in grafts that did not survive (Figure 5).

### Neural- and retinal-specific markers in grafts

**Figure 6:**
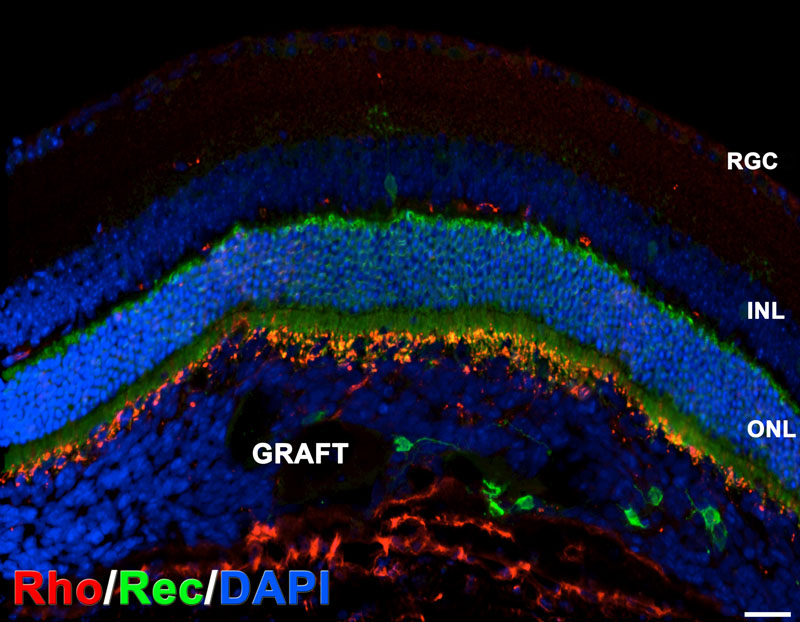
Absence of rhodopsin and scarce presence of recoverin-positive human cells in grafts at 3 weeks after transplantation. There were no rhodopsin-positive cells in neural grafts at this time point, although rhodopsin staining was present in the outer segments of the host retina as expected. Only few recoverin-positive cells were identified in subretinal grafts at this time point. The abbreviations used in this figure were the following: RGC, retinal ganglion cells; INL, inner nuclear layer; ONL, outer nuclear layer. The scale bar used in this figure represents 50 μm.

**Figure 7:**
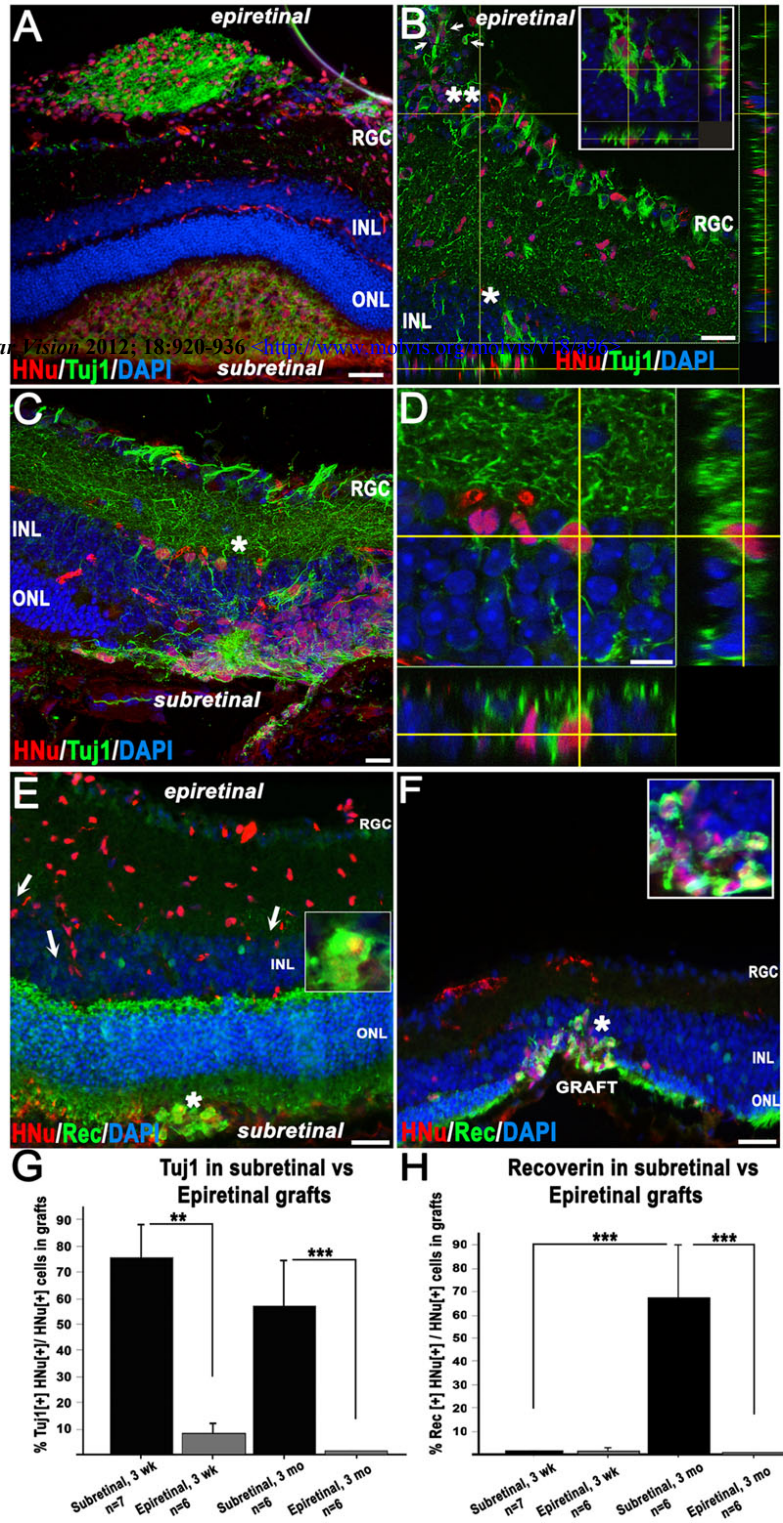
Differences in retinal integration and maturation of human embryonic stem cell-derived retinal progenitor cells (hESC-RPCs) transplanted into subretinal and epiretinal space. Confocal images in panels **A**-**F** represent 3-month grafts. The majority of cells in epiretinal grafts (except for some rare larger human nuclei – positive, Tuj1 – positive (HNu [+] Tuj1 [+]) clusters (shown in panel **A**) or HNu [+] Tuj1 [+] cells found in the host’s retinal ganglion cell (RGC) layer (**) or inner nucler layer (INL) (*) but not inner plexiform layer (panel **B**) do not display the Tuj1 marker. The scale bar in panel **A** represents 50 μm. **B**: Many HNu [+] cells, including HNu [+] Tuj1 [+] neurons, were found embedded into the host’s INL (*) and RGC layer (**). White arrows point to HNu [+] Tuj1 [+] neurons in the host RGC layer. Inset in **B** is a high-power *z*-stack confocal image of the INL area shown with an asterisk (*), which has several HNu [+] Tuj1 [+] neurons. The scale bar in panel **B** represents 50 μm. **C**: Migration of subretinally grafted cells into the host’s NR when the retinal architecture was damaged by injection. **D**: *Z*-stack confocal analysis of the area shown in **C** with an asterisk, demonstrating the HNu [+] Tuj1 [+] neuron integrated in the host’s INL. **E**: The difference in acquisition of photoreceptor marker recoverin by subretinal grafts versus epiretinal grafts at 3 months following grafting. While the human cells located in the subretinal grafts display predominantly recoverin-positive immunophenotype (recoverin [+]), the human cells in the epiretinal grafts remain recoverin-negative (recoverin [-]) and slowly migrate into the RGC and INL layers. White arrows show the elongated human nuclei, typical for migrating cells. The scale bar in panel **E** represents 50 μm. **F**: Integration of recoverin [+] human cells from subretinal grafts into the host’s PR layer when the host retina was damaged during injection. The scale bar in panel (F) represents 50 μm. **G**: Different dynamics of loss of immature neuronal marker Tuj-1 in subretinal and epiretinal grafts. In the subretinal grafts (black bars), the reduction of the number of HNu [+] Tuj1 [+] cells in grafts in time from ~76% (3 week, n=7) down to ~57% (3 months, n=6; grouped for both hESC lines) is due to the maturation of cells to a recoverin [+] developmental state (shown in **H**). In the epiretinal grafts (gray bars), the majority of cells (more than 92% at 3 weeks and about 99% by 3 months) are Tuj1 [-], with many cells being nestin - positive (not shown). Values represent the mean percentage of HNu [+] Tuj1 [+] cells in human grafts±standard error of the mean (SEM); **, p<0.005; ***, p<0.0005. The percentage of HNu [+] Tuj1 [+] cells in subretinal grafts was statistically higher compared to that in epiretinal grafts, both at 3 weeks and at 3 months. There was a trend for reduction of the percentage of HNu [+] Tuj1 [+] cells in human grafts at 3 months, compared to that at 3 weeks, but the difference was not statistically significant. **H**: The dynamics of acquisition of the photoreceptor marker recoverin in subretinal and epiretinal grafts. There are no differences in the number of recoverin [+] human cells (less than 1% HNu [+] recoverin [+] cells) in subretinal and epiretinal grafts at 3 weeks following grafting. However, while cells in the epiretinal grafts continue to be recoverin [-] at 3 months following transplantation, cells in the subretinal grafts mature to a recoverin [+] immunophenotype (about 67.5%, n=6). Values represent the mean percentage of HNu [+] Rec (recoverin) [+] cells in human grafts±SEM; ***, p<0.0005.

Grouped data for both hESC lines showed a reduction of immature neuronal marker Tuj1 in 3-month subretinal grafts (57.2% Tuj1 [+] hESC-RPCs [n=6], Figure 2E-G) compared to that at 3 weeks (75.7% Tuj1 [+] hESC-RPCs [n=7], Figure 2B, also see the plotted graph in Figure 2D). Further maturation of subretinally located hESC-RPCs was evident as only 1.3% hESC-RPCs were recoverin [+] at 3 weeks (n=7; Figure 2C and Figure 6) whereas at 3 months 67.5% were recoverin [+] (n=6; Figure 2I,J, also see the plotted graph in Figure 7H). Approximately 15% of grafted cells were mitotically active at 3 weeks (n=7), but only a few HNu [+] cells were stained with proliferation marker Ki67 by 3 months (less than 0.01%, data not shown). No tumor formation was observed in grafts. Human-specific synaptophysin [+] sparse human boutons resembling *boutons en passant* were found both on Tuj1 [+] axons emanating from the grafted HNu [+] cells (Figure 2K, inset) and on host PRs. In a few grafts (at 3 months only), cells were positive for rhodopsin (Figure 2L).

### Differentiation and migration of cells in subretinal versus epiretinal grafts

Substantial differences were found in maturation of hESC-RPCs in subretinal versus epiretinal grafts (Figure 7). Subretinal grafts demonstrated a little decrease of Tuj1 immunostaining (from about 75.7%, [n=7] at 3 weeks to 57.2% [n=6] at 3 months). However, the difference was not statistically significant at p<0.05 when combined for both TE03 and UC06 cells. Three-week-old epiretinal grafts had less than 8% of Tuj1 [+] cells. By 3 months, only about 1% of cells in the epiretinal grafts were HNu [+] Tuj1 [+], and these were mostly in small clusters (Figure 7A). However, cells from epiretinal but not subretinal grafts were able to easily integrate into the host’s RGC and INL layers even when host retina was not damaged (Figure 2H, Figure 7A,B,E). Cells from the subretinal grafts were detected in the ONL (and rarely in the INL) only when the host retina was damaged (Figure 2F and Figure 7C,D,F). IHC with recoverin and HNu antibodies demonstrated a sharp increase in the number of HNu [+] Rec [+] cells in subretinal grafts from about 1% at 3 weeks [n=7] to about 67.5% at 3 months [n=6] (combined for both TE03 and UC06). Cells in the epiretinal grafts displayed a low presence of recoverin [+] human cells at 3 weeks (less than 2%), and no such cells were present by 3 months. Note the complete absence of HNu [+] recoverin [+] cells in the RGC/INL and clusters of HNu [+] recoverin [+] cells in the subretinal space (Figure 7E). qRT–PCR analysis corroborates this data and shows that early progenitor/PR markers (*RCVRN* [recoverin], *MASH1* and *NEUROD1*) are upregulated at the time of grafting. Evidently, such cells could undergo further maturation in the subretinal but not epiretinal niche.

## Discussion

Stem cell-mediated cell replacement therapy for retina has advanced rapidly in the past several years and has the possibility of becoming a treatment method for some retinal degeneration (RD) conditions [35,36,56]. Apart from reproducibility of the data from different hESC lines, many issues require further evaluation; these include OLM barrier [23,26,57,58], immunorejection of graft by a host [27,59] (excellent discussion in [60]), and the formation of glial scar containing extracellular matrix and Müller glia endfeet, preventing further cell integration [58,61]. In addition, the host retinal niche and preservation of retinal architecture of the recipient seem to contribute to the complexity of any graft’s survival and functional integration [24].

We considered it important to investigate two separate recurrent questions frequently reported in retinal cell transplantation papers: the survival of the retinal grafts in a non-immunocompatible recipient and the population of retinal layers with grafted hESC-RPCs. We approached this by first selecting normal (non-RD) young adult mouse eyes as recipients of hESC-RPC grafts to avoid the influence of a degenerating and rapidly changing neural niche on the survival of the graft [24,62-64]. Such reports, although debated, suggest that an injured or degenerative neural environment might adversely affect the survival of human stem cell-derived grafts. We also chose not to apply immunosuppression, as retina is considered an immunoprivileged site due to the blood-retinal barrier (BRB). In addition, survival of xenogenic human grafts in retina has been reported [50]. To account for the expected differences in graft survival, we correlated the survival of transplanted cells with the overall integrity of the RPE/choroid tissue, which comprises the BRB [65]. Lastly, we compared the dynamics of cell integration into the host’s retina from the subretinal and epiretinal space to circumvent the OLM barrier. The advantage of such an approach is that in any given transplantation case the grafting niche remains the only difference, which may be informative for data interpretation. Overall, we find that both hESC lines UC06 and TE03 (cultured for 50+ passages) can differentiate to mature retinal phenotypes using the noggin/Dkk-1/IGF-1/bFGF/FGF9 protocol. After 3 months in a subretinal environment, transplanted cells demonstrated the ability to acquire mature PR-specific immunophenotypes (e.g., recoverin and rhodopsin staining) and no tumorigenicity was detected in all examined grafts. Importantly, we observed that the survival of xenogenic grafts with no immunosuppression correlates with the integrity of the RPE/choroid structure (BRB) but not the NR. Whenever the histology showed no damage to the RPE/choroid, the graft survived and thrived for up to 12 weeks with no immunosuppression and no signs of deterioration. The damage to the host’s NR alone and/or strong activation of GFAP by reactive Müller glia of the host (Figure 2 and Figure 3) did not affect graft survival. In cases when the RPE/choroid showed signs of substantial damage by a blunt needle guided by the nano-injector, xenogenic hESC-RPC grafts did not survive, displayed lysed human cells, were filled with host’s Iba-1 [+] microglia, and were GFAP [+]. Therefore, we conclude that the xenogenic grafts may survive and thrive in the subretinal space when the BRB is intact. Consequently, systemic immunosuppression may not be necessary for graft survival when nonautologous PR progenitors are transplanted into retina.

Our results showed limited integration of subretinally grafted hESC-RPCs into the host’s retina and only in cases when the ONL had some structural damage. However, no HNu [+] cells (except one case) were found in INL or RGC layers, likely due to intact OLM present in the wild-type retina, consistent with other reports [19,23,57]. In contrast, integration of hESC-RPCs into the host INL and especially the RGC layers was efficient from the epiretinal grafts, irrespective of whether the retina had any structural damage. Some HNu [+] cells were co-localized with host RGCs and also expressed RGC marker Tuj1 [66]. qRT–PCR analysis of cells at the time of grafting showed that hESC-RPCs upregulated RGC markers (such as *MATH5* and *BRN3B*) and the horizontal neuronal marker (e.g., *PROX1*). Thus, hESCs could potentially generate RGCs and horizontal cells.

A limited number of human synaptophysin [+] boutons en passant could be detected in the INL and RGC layer, indicating initiation of synaptogenesis. Due to the lack of a barrier for cell penetration from the epiretinal side, such grafts may be used for long-term trophic support of degenerating retina [67], including the trans-synaptic transport of neurotrophins [68], as well as for potential RGC and INL cell-replacement strategies. Although the migration of cells into the ONL from subretinal grafts was clearly impeded, we suggest that in RD conditions this migration could be helped by a porous OLM [69] as well as guided by tropism of grafted progenitor cells to the sites affected by degeneration [5,70]. It is also possible that the maturation state of hESC-RPCs affects integration as some studies have reported integration of postmitotic progenitors and even mature PRs into normal retina [35,71]. Although immunosuppression may not be crucial for xenogenic graft survival, it may be beneficial for retinal integration in a clinical setting when nonautologous (i.e., stem cell-bank-derived) hESC-RPCs are transplanted subretinally. For example, removal of glial barrier in GFAP^−/−^ and vimentin ^−/−^ mice provided a permissive environment for retinal integration of transplanted neurons [31]. Such a glial barrier, induced by the host, may be partially alleviated by immunosuppression and chondroitinase ABC [61].

We also noted that the subretinal but not the epiretinal niche can provide further cues for hESC-RPC maturation to PRs, resulting in a sharp gain of mature PR marker recoverin, a neuronal calcium-binding protein found almost exclusively in PRs [16]. However, the epiretinal grafts demonstrated no cell maturation and retained the original, mostly nestin [+] immunophenotype. This is consistent with a previous observation [49] indicating that paracrine morphogens in the host retina and/or RPE can promote further maturation of hESC-RPCs.

As the cell population at the time of grafting showed almost 100% neuralization with noggin and over 67% of cells in grafts were positive for PR marker recoverin by 3 months, the overall efficiency of PR-fate specification from both hESC lines appears to be comparable to that reported [16]. Only a small number of cells neuralized by noggin may be expected to remain non-neural after 4 weeks in culture [53]. Since only neural rosettes were collected for further induction with Dkk-1 and IGF-1, the number of non-neural cells in such cultures should be minimal by day 50 (grafting), thus reducing tumorigenicity. There are several important distinctions resulting in faster derivation of recoverin/rhodopsin immunophenotypes in cultures reported earlier [16]. These differences potentially originate from somewhat longer exposure to Dkk-1 and IGF-1, culturing on Matrigel rather than defined gelatin/laminin coating, and likely different culturing densities, which may profoundly influence the dynamics of neuronal cell fate acquisition and maturation [72-74]. We also chose to maintain both bFGF and FGF9 in neural cultures, which earlier received anteriorizing Dkk-1 and IGF-1 induction, as both bFGF [75] and FGF9 [76] reportedly bias early retinal cells to an NR rather than an RPE cell fate.

The influence of FGF9 on the NR versus RPE cell fate is especially interesting as it is unexplored in retinal differentiation protocols. *Fgf9* is expressed in the distal part of the developing optic vesicle in the mouse that is destined to become a NR and was reported to induce activation of *Ras* by receptor tyrosine kinase in early optic neuroepithelium [76]. Ectopic expression of *Fgf9* in the proximal region of the optic vesicle destined to become RPE promotes conversion of the RPE cell fate to an NR cell fate in early retinal development by suppressing the expression of RPE marker *Mitf* and induction of NR-specific markers *Rx*, *Chx10,* and *Atoh7* (*Math5*) [76]. As a result of such ectopic expression, a duplicated NR has been produced. Notably, the original NR and duplicated NR differentiated and laminated symmetrically but with a mirror-image polarity. The same study delineated the likely downstream target of FGF9 signaling, promoting the acquisition of the NR cell fate: the RAS-mediated RAF-MEK-mitogen-activated protein kinase pathway. Specifically, the transient expression of a constitutively active human *Ras* oncogene by tyrosinase-related protein2 (TRP2) promoter in mouse transgenic embryos also converted the developing RPE to a second NR. Because the retinal development in both types of transgenic mice was overall normal, it was concluded by Zhao et al. [76] that FGF9 signaling was needed to define the boundary between the retina and the RPE. Collectively, transient FGF9 signaling, likely through RAS signaling, was sufficient to promote NR cell fate at the expense of RPE, which was one of the goals of our differentiation protocol. Other factors, such as ectopic *Pax6* expression or null mutation of *Chx10,* are known to shift the cell fate in the developing retina from RPE to NR and vise versa, respectively. However, such signaling requires genetic manipulations in hESCs compared to easy delivery of FGF9 (and bFGF) morphogens during the differentiation protocol.

FGF9 belongs to a different subfamily of FGF factors compared to bFGF (FGF2) and can inhibit the canonical *Wnt* pathway via upregulation of *Dkk-1*, a canonical *Wnt* antagonist, and regulate the transcription of Hedgehog targets patched homolog 1 (*Ptch1*) and glioma-associated zinc finger 1 (*Gli1*) independently of the Hedgehog ligand [77]. Both effects may promote NR differentiation [16,78]. Additional investigations are necessary to clearly delineate the role of FGF9 in NR differentiation.

In summary, we show that (i) xenogenic human hESC-RPC grafts from both hESC lines survive in the subretinal space without immunosuppression when little structural damage occurs to the RPE/choroid; (ii) gradual maturation of hESC-RPCs in subretinal but not epiretinal grafts occurs over a period of 3 months, indicating that the subretinal but not the epiretinal (vitreous) niche provides further differentiation cues for retinal cell fate maturation; (iii) substantial migration and integration of hESC-RPCs into the RGC and INL layers from epiretinal grafts occurs, even when the host retina lacked signs of damage. Our data provide new insights into differentiation and integration of grafted cells and may advance the protocols for cell therapies of retinal degenerative diseases.
